# Point-of-care ultrasound in critically ill COVID-19 patients: questions derived from practice

**DOI:** 10.1186/s13089-021-00254-2

**Published:** 2022-01-03

**Authors:** Pablo Blanco

**Affiliations:** 1 High-Dependency Unit/Critical Care COVID-19 Unit (UCIM), Hospital “Dr. Emilio Ferreyra”, 4801, 59 Ave., 7630 Necochea, Argentina; 2 Department of Teaching and Research, Hospital “Dr. Emilio Ferreyra”, 4801, 59 Ave., 7630 Necochea, Argentina

**Dear Editor,**

I read with interest the proposal of Soldati et al. [1] regarding the use of lung ultrasound in COVID-19 patients. They aimed at optimizing resources and time, reducing personnel exposure and avoiding environmental viral spread, while guaranteeing a high-quality ultrasound study. However, over time, after facing with several coronavirus waves, many of these recommendations may be questioned, particularly the type of machine used, the need of covers and the number of operators required. The objective of this letter is discussing the aforementioned issues, pointing toward the need for updates of recommendations.

## First, the type of machine used

Soldati et al. [1] proposed the use of a wireless ultrasound unit for lung ultrasound, while others used non-wireless devices [2]. The use of pocket-sized machines and wireless probes are encouraged, because these are easy to clean, transport, and disinfect, and therefore, may aid in limiting equipment contamination and viral spread [1, 3–6]. While useful for lung ultrasound and for mean patients, the image quality and capabilities of these devices are inferior compared to the more powerful machines typically used in the ICU, features that in fact are needed in many critically ill COVID-19 patients (e.g., for measuring the cardiac output, for measuring parameters of venous congestion and so on). Regardless the size of the device, and given that coronavirus persists in inanimate surfaces [7], a careful equipment cleaning and disinfection should always be done for any equipment used. Quaternary ammonium compounds are compatible with most ultrasound machines and is highly effective against COVID-19 [8]. Therefore, from a practical point-of-view, there is no need of advocating the use of miniaturized devices for infectological reasons providing the equipment is carefully cleaned and disinfected. Similar to others [2], we maintained the image quality and capabilities of always using a conventional portable machine (Mindray M6®) dedicated to the COVID-19 ICU and equipped with three transducers: convex (2–5 MHz), phased-array (2–4 MHz) and linear (5–10 MHz) probes.

## Second, the need of covers

Covering the ultrasound equipment, transducers, cables and cords, is recommended to minimize equipment contamination/viral spread and to ease equipment cleaning and disinfection [1, 3–6]. Intuitively, the smaller the device, easier covering it.

However, in practice, covers are not so often available, their use may be time consuming, and may even lower the image quality. Also, covering the machine with a large clear drape may produce unproper equipment functioning because of overheating [9]. Given that equipment cleaning and disinfection must be always performed as stated before, the use of covers sounds controverted. Therefore, using covers seems needless and so many centers like ours avoid using them routinely (except for sterile procedures such as midline or central venous cannulations).

## Third, one or two operators (or none)

In the proposal of Soldati et al. [4], authors recommend that two operators perform point-of-care lung ultrasound using a pocket device composed by a wireless probe and a tablet. One operator manipulates the transducer and a second manipulates the tablet and selects, freezes and stores images. The second operator may be in the isolation room separated at least two meters from the patient or may even remain outside the isolation room, communicating with the first operator by a phone call if needed. Although not validated in the literature, authors advocate that this practice minimizes the first operator’s exposure or dependence, while minimizes or avoids exposure of the second. In our view, all these sounds doubly, time-consuming, useless and do not avoid the exposure of one operator at least. In addition, in many countries such as Argentina, finding two intensivists with competences in lung ultrasound (and in POCUS in general) sharing the same ICU is uncommon. Also, POCUS is more than just lung ultrasound, and more powerful devices are typically needed. Therefore, full body POCUS (including lung ultrasound) performed by a skilled operator using a conventional ultrasound machine seems more reasonably. Anecdotical experiences using robotic ultrasound have been described by colleagues [10, 11] aiding in completely avoiding operator exposure; however, although promising, this technology is expensive and not widely available. Therefore, in practice, operator exposure seems unavoidable and therefore, full personnel protective equipment should always be used.

In conclusion, early recommendations about the use of POCUS in critically ill COVID-19 patients can be questioned, and probably needs to be revised and simplified. These updated recommendations may be useful to physicians facing with a next coronavirus wave or for those who lack expertise yet working with ultrasound in the pandemic.

## Data Availability

Not applicable.
